# 

**Published:** 1877-03

**Authors:** 


### INDEX TO VOLUMME XIV.

Abscess of Anus in Typhoid Fever.......... 41
Acute Dysentery, Sulphite of Soda in.......... 42
Acute Rheumatism, Compression in.......... 55
Alcohol, Does it afford Nutrition to the Animal Tissues.......... 65
ALLEY, A. R.......... 73
Atlanta Academy of Medicine, Proceedings of..12, 91, 153, 213, 264, 347, 390, 454, 540, 587, 660, 719
An Apology.......... 186
A Plea for Principles and Conservatism in the Treatment of Diseases Peculiar to Females, etc.......... 193
American Medical Association, Twenty-second Annual Meeting.......... 249
An Inch Screw Swallowed by a child two years old.......... 264
American Dermatological Association.......... 315
Pharmaceutical Association 459
ARNOLD, Dr. R. D. of Savannah, 316
Air as an Anæsthetic.......... 354
An Interesting Family.......... 383
An Introduction to Pathology and Morbid Anatomy.......... 439
A Treatise on the Science and Practice of Midwifery.......... 439
A new Principle in the Application of the Obstetric Forceps.......... 476
Artificial Diamonds.......... 510
Ambergris.......... 512
Association of American Medical Colleges.......... 564
Amputation of Arm by Means of the Elastic Ligature.......... 634
Absence of the Uterus, etc.......... 687
Almen's Test for Blood.......... 702
Bi-Lateral Lithotomy, two cases of.......... 10
Bacteria found in Perspiration of Man.......... 38
BAIRD, JAS. B.......... 1
BEAUCHAMP, J. C.......... 152
Bed-Bug or No Bed-Bug.......... 358
Brain Lesions.......... 368
BATTEY, ROBERT.......... 385
Blood-letting in Puerperal Eclampsia..........438, 449
Burns, Treatment of by White-lead.......... 566
Bougies of Gelatine.......... 172
BRAGG, SHIRLEY.......... 645
BOOZER, W. D.......... 715
COLEMAN, J. F.......... 10
Ciliary Neuralgia, Cure by Quinia.......... 20
Cataract, Traumatic, with Iritis and Secondary Cataract.......... 24
CALHOUN, A. W..........20, 321
Cough.......... 34
Constipation and Diarrhœa, Treatment of.......... 39
Castor Oil, Agreeable Mode of Taking.......... 46
Chronic Disentery, Treatment of.......... 52
Calculi in Prepuce of Child.......... 54
Compound Urinary Fistula, case of.......... 83
Chloral in Pityriasis.......... 111
Case of Tænia Solium Simulating Pleuritis.......... 152
Child Crying in Utero..........152, 380
Clinic Atlanta Medical College, 167
Circulation Disturbances in Traumatic Lesions of Brain, 182
Caustic in Treatment of Diseases of the Neck of Bladder, 183
Catalogue of National Medical Library.......... 184
Convention of Medical Colleges..........187, 312
Correction.......... 192
"Calami ies of Surgery".......... 235
Cyclopædia of Practice of Medicine..........240, 373, 701
Concerning the Inoculability of Typhoid Fever.......... 279
Camphor Poisoning.......... 286
Cod Liver Oil as a Therapeutic Agent.......... 291
Chloral in the Pains of Parturition.......... 296
Can Tuberculous Matter, Taken as Food, excite Tub[illegible]lous Diseases?.......... 29[illegible]
CAMPBELL, A. S.......... 513
Convulsions, Arrest of by the Sinistro Lateral Posture.......... 301
Cholera Morbus.......... 303
Cataract Operations, Report of Sixty-nine.......... 321
Capillary Nævus, Treatment of, 366
Cearbro-Spinal Meningitis, Pathogomonic Symptoms of.......... 373
Chemistry.......... 436
Communication to the Medical Association of Georgia.......... 494
Chronic Nasal Catarrh.......... 552
Cincinnati Academy of Medicine.......... 674
Clinical Reports.......... 676
Caloropathy.......... 692

Case of Full Term Extra Uterine Gestation of Tubo-Ovarian Form.................. 135
Of Tetanus Treated with Calabar Bean........... 621
Of Urethritis from Constricted Meatus......... 651
Ceribro-Spinal Meningitis..... 712
Cutaneous Diseases, Sand-Friction in .................... 754
Diptheria..................35, 567
Drunkenness, Rusian cure for, 41
Digitalis in Bronchitis........ 51
Diseases of Infancy and Childhood ...................... 57
Death of Dr. Nottingham..... 63
Dr. Ralph Walsh's Call-Book, 128, 498
Dean, W. H.................. 153
Diminution of Spleen under Hypodermic use of Ergot... 177
Damiana..................... 183
Dermoid Cyst of Ovary....... 211
Dysmenorrhœa, Remedy for... 280
Diseases of the Nervous System ......................... 309
Of the Eye ......... 437
Of the Pancreas.... ..... 466
Determination of Sex......... 365
Differences between the Anæsthesia of Ether and Chloroform ...................... 370
Dermatology................. 502
Description of an Epidemic of Dengue ...................... 596
Drainage of Wounds, New Plan for .......... .......... 629
Dandruff, Remedy for......... 632
Dangers of the Pessary....... 639
Ear. Suppurative Inflammation of, with Necrosis of Mastoid Process.................... 22
Removal of Foreign Bodies from .................. 113
Extirpation of Larynx and Part of Tongue................. 44
Ergot, Its active Principles... 49
In Parturition............ 88
In Treatment of Glactowhœa and Mastitis....... 181
Hæmoptysis, Treated by.. 373
On the Post Partum use of in Obstetrical Practice.. 705
Extra Uterine Pregnancy...... 58
Enteric Fever, Turpentine Treatment in.............. 114
Esmarch's Method Simplified.. 183
Bloodless Oparation,....... 263
European Correspondence, 186, 439, 758
Editorial Notes............... 241
Etherization................... 287
Ether, Death following use of.. 435
Epitome of Skin Diseases..... 570
Errata....................... 576
Editorial Correspondence. 625, 697
Epilepsy from Disease of Internal Ear.................... 717
Foul Breath, Salicylic Acid in.. 45
Fever, Application of Quinia in......................... 282
Lectures on ............. 439
Flint River Medical Association 319
Fracture of the Clavicle....... 454
Goitre in Liberia............ 47
Gleanings ................58, 116
Georgia Medical Association.. 119
Gouley, J. W. S............. 257
Gelseminum Sempevirens..... 295
In Neuralgia............. 508
Gonorrhœa, Injection of Quinine in..................... 296
Glycerine as a Therapeutic Agent ..................... 368
Griggs, J. W................ 579
Hydraphobia, Pathology of.... 49
Heart Clots and Embolism, and Esmarch's Bandage in Amputation......... ...... . 74
Hospital Reports, New York Woman's Hospital.......... 108
Hypodermic Injections, Where to make.................... 113
Use of the Tincture Veratrum Viride in Puerperal Eclampsia ............. 579
Injections of Carbolic Acid in Neuralgia........... 600
Heard, G. B................. 133
Human Physiology, Text-Book of ......................... 241
Herniotomy for Reducible Inguinal Hernia.............. 360
Hopospadias, Treatment of.... 365
Human Physiology, Principles of ......................... 571
Infantile Eczema, Lime Water in......................... 32
International Medical Congress, 62, 314, 393
Irritability of the Bladder, treated by Dilatation of Urethra.. 116
In Memorium................ 124
Inflammation and its Curability, Remarks on............. 129
Intestinal Obstruction........ 175
International Medical College. 190
Impudence.................... 191
International Metric System of Weights and Measures...... 498
Instantaneous Sinapisms...... 510
Imperforate Hymen, the Cause of ........................ 560
Johnson, Jno. Thad ......210, 577

JOHNSON, J. M. .................. 449
Jaundice, Cause of............ 504
KENDRICK, W. S. .............. 26
Lunacy in Town and Country. 55
Lister's Treatment of Wounds by the Antiseptic Method... 98
Lister's Antiseptic Method in Ovariotomy.................. 630
LOVE, WM. ABRAM.........193, 705
Lectures on Orthopedic Surgery and Diseases of Joints... 305
Latent Gonorrhœa............ 631
Luxation of Both Shoulders .. 634
Muscles of Neck, Contraction of 12
Midwifery, System of......... 57
Medical Association of Georgia 64
Mode of Applying Iodide to the Interior of Cysts............ 106
Manual of General Pathology.. 118
Of Percussion and Auscultation .................. 436
Medical Thermometry and Human Temperature......... 118
Clinic Atlanta Med. College 592
Muscular Exertion as a Cause of Albumenuria ........... 184
Michigan University of Homœopathy .................... 191
Monthly Abortions............ 305
Micro Photographs in Histology, Normal and Pathological 310, 377, 571
Medical and Surgical Memoirs, 374
Measures and their Equival'nts, 511
McINTOSH, T. M. ............. 535
Maternal Impressions Affecting the Fœtus ................. 549
McCLELLAN, ELY............. 582
Medical and Surgical Society of Baltimore...... .......... 588
Modern Turkish Bath ........ 652
Notes for Examina ion of Class 26
Of Practice, Bellevue Hospital.................... 101
Nervous Diseases, Deaths from 33
New Method of Preventing Secretion of Milk in Female Breast..................... 37
Nelaton's Catheter, Use of..... 109
NewYork Academy of Medicine 178
Nervous Aphonia Cured by Inhalation of Chloroform...... 181
"No More Ovariotomy"...... 182
Nerve-Stretching in Tetanus .. 364
Lesions as the Origin of Certain Skin Diseases... 532
New York Observer........... 576
Nitro-Glycerine in Epilepsy... 632
Notice of Dissolution......... 762
Ovarian Tumors, Cure of by Electrolysis...... ......... 43
Ozœna, Treatment of......... 54
Opium Poisoning, Case of .... 73
"Antidote".............. 757
Obstinate Neuralgia, Pills for.. 116
Oxygen Gas a Remedy in Disease.................... 133
On the Employment of Cold .. 285
Ovariotomy, Extraordinary Success in ................ 299
Orchitis, Treatment of........ 309
Ovarian Cysts, Treatment of by Drainage .................. 365
Oxygen Gas................... 368
Obesity Treated by Sea Water, 511
Otology, Recent Progress in... 555
Ozone Observations in Paris... 640
Obstetrics, Remarkable Case of 753
Osteotomy in "Gum Valgum" 756
Prevention of Pain after Actual Cautery .................. 43
Poisoning by Chloral, Antidote 46
Piles, Radical Cure for ....... 56
Phthisis...................... 58
Pruritis...................... 184
Pamphlet Notices....185, 310, 377
Phimosis, New Method of Performing Circumcision for ... 210
Proceedings of Convention of American Medical Colleges.. 243
Post-Diphtheritic Paralysis, Case of.................... 277
Pharr, Dr. Edward........... 319
Poison-Oak Eruption......... 363
Prolapsus Ani in Children, Treatment of................ 367
Puerperal Scarlatina.......... 426
Convulsions.............. 602
Pathology and Treatment of Child-Bed ................ 439
Poisoning by Gelsemium...... 496
Pepsin ...................... 497
Poisonous Properties of Glycerine..................... 509
Prolapse of the Iris .......... 535
Post-Partum Hemorrhage..... 568
Poisonous Principle of Spoiled Corn...................... 575
Prayer and Medicine.......... 628
Pleural Effusions.... ........ 632
Practical Treatise on Diseases of the Skin ................ 700
Quinetum and its Sulphate.... 633
Results of Operations on Scrofulous Persons.............. 47
ROSSER, L. P ................ 211
Rheumatic Fever, Treatment of with Sylicilic Acid.......... 283
Rheumatism, Salicylic Acid in, 359
Raw Onions as an Hypnotic... 367
Rheumatism, Salicin and Salicylic Acid in............... 369
Review of Woodhull's Non-Emetic Use of Ipecacuanha.. 582

Regeneration of Nerves after Neurotomy ............... 636
Rupture of the Uterus, Case of 478
Spine, Potts' Disease of...... 31
Sore Throat, Common Membranous .................... 36
Skin Diseases, Petroleum in... 42
Specula for Examining Female Bladder ........... ........ 53
Suppurative Pericarditis, Case of........................ 55
Surgery, Treatise on.......... 56
Stevens, J. P................ 65
Shafer, A. J................. 88
Sunstroke, Pathology of...... 105
Sequel to the Case of Habitual Constipation................ 110
Systemic Infection from Purulent Vaginal Discharges ... 112
Starr, E. F.................. 129
Stone in the Bladder ......... 257
Stanford, F. A............... 263
Section of United Fracture and Wiring of the Ends ........ 284
Salicylic Acid as a Febrifuge.. 292
Syphilitic Buboes, Treatm'nt of 293
Syphilis, Is it ever a Cause of Phthisis?.................. 294
Scientific Careers............. 297
Sulphate of Quinia as an Excitant of the Uterus........... 363
Semi-Malignant Mammary Tumors, Value of Pressure in.. 371
Skin Diseases, Atlas of........ 376
Salicylic Acid................ 379
Precautions with the Use of 639
Sulphate of Cinchonidia...... 385
Salutatory.................... 446
Splenetic Leucocythæmia..... 481
Small-Pox, Salicylic Acid in .. 508
Solution of India-Rubber in Local Treatment of Psoriasis 576
Salicylate of Ammonia as a Substitute for Salicylic Acid..... 576
Stricture of Urethra, Narrowed Meatus as a Cause of........ 577
Surgery, Recent Progress in... 614
State Board of Health........ 623
Santonine, Dangers from...... 629
Stricture of the Male Urethra.. 641
Septicæmia................... 680
Secondary Hemorrhages...... 729
So-Called Ulceration of the Womb ..................... 734
Secondary Hemorrhages ...... 729
Trenton Medical Association.. 39
Taliaferro, V. H............. 83
The Most Useful Drugs....... 282
Transactions of State Association ...................... 192
Medical Association of Alabama................. 381
The Enemata of Wine........ 281
Treatment of Epistaxis by internal administration of Ergot........... ........... 288
Treatment of Cystitis by Atropia Enemata............... 295
Tetanus following Hypodermic Injection of Quinine........ 298
Transferance of Measels to Dogs 368
Therapeutics, Treatise on..... 376
Transactions of Texas Medical Association..... .......... 384
The Scourge.................. 447
True Site and Probable Cause of Placenta Prævia......... 484
Tooth Powder................ 512
The Ovalution Theory of Menstruation .................. 569
Mad Stone................ 573
Eucalyptus in Ague...... 574
Metric System in Prescriptions .......... ...... 620
Treatise on Practice of Medicine ...................... 570
Traumatic Scepticæmia........ 715
Tetanus, Forcible Extension of Nerves in....... 755
Uterine Hemorrhage.......... 50
Urinary Organs............... 437
Urine, Analysis of............ 701
Ulceration of the Womb ...... 734
Viscera, Diseases of.......... 180
Valedictory .................. 448
Vaginal Ovariotomy.......... 569
Violent Pain, Its Effect on Animals and Men.............. 638
Whooping Cough, Decoction of Chestnut Leaves in...... . 14
Abortive Treatment of with Sulph. Quinine......... 506
Wall, John P................ 74
When we may and when we may not bleed.............. 288
Whooping Cough............ 289
Watson, J. C................ 454
Westmoreland, W. F......... 448
Westmoreland, J. G......447, 712
Westmoreland, R. W.....447, 532, 651, 717
Whisky in Tetanus.......... 628
Wilson, J. Stainback......... 652
Yellow Fever... ............ 491
Is the Cause Exotic or Indigenous?.............. 693

